# Retraction Note: *Astragalin* alleviates cerebral ischemia-reperfusion injury by improving anti-oxidant and anti-inflammatory activities and inhibiting apoptosis pathway in rats

**DOI:** 10.1186/s12906-023-03906-z

**Published:** 2023-03-07

**Authors:** Xiuying Chen, Chang Cheng, Xuzheng Zuo, Wen Huang

**Affiliations:** 1 Department of Neurology, Second Affiliated Hospital of Army Medical University, No.83 Xinqiao Main Street, Shapingba District, Chongqing 400037, China; 2 Department of Neurology, General Hospital of southern Theatre Command, Liuhua Road, Guangzhou 510010, China

**Retraction Note: BMC Complement Med Ther 20, 120 (2020)**

10.1186/s12906-020-02902-x

The Editor has retracted this article at the request of the corresponding author because the authors have been unable to repeat the results. In addition, there appears to be significant overlap with another publication [1]. Wen Huang agrees with this retraction. None of the other authors has responded to correspondence from the Editor about this retraction.
